# Motives for participating in a clinical research trial: a pilot study in Brazil

**DOI:** 10.1186/1471-2458-13-19

**Published:** 2013-01-10

**Authors:** Solange A Nappo, Giovanna B Iafrate, Zila M Sanchez

**Affiliations:** 1 Brazilian Center of Information of Psychotropic Drugs (CEBRID), Department of Preventive Medicine, Universidade Federal de Sao Paulo, 04038-034, Sao Paulo, Brazil

## Abstract

**Background:**

In the past, clinical study participants have suffered from the experiments that they were subjected to. Study subjects may not understand the study process or may participate in clinical studies because they do not have access to medical care. The objectives of the present study were 1. to analyze the motives that might cause a volunteer to participate as a study subject; 2. to identify the social-demographic profile of this study subjects; and 3. to determine whether the motives to volunteer as a study subject are in accordance with the established legal and ethical principles for research in Brazil.

**Methods:**

Mixed-methods research was used (a qualitative-quantitative approach). A sample of 80 volunteers underwent a semi-structured interview, which was based on a survey script that was elaborated from discussions with key informants. The sample was randomly selected from a database of clinical study volunteers that was provided by Brazilian clinical study centers. The interviews were recorded and transcribed. Descriptive statistics were used for content analysis, including contingency tables with hypothesis testing.

**Results:**

The motivations for clinical study participation were linked to types of benefit. The most frequently encountered motivations were financial gain and therapeutic alternative. Altruism was not a common motivator, and when altruism was present, it was observed as a secondary motivator. All participants reported that they understood the Informed Consent Statement (ICS). However, only two parts of the form were remembered by all of the volunteers: the section on being able to leave the study at any point and the section that stated that there would be some responsible professional at their disposal for the entirety of the study.

**Conclusions:**

The present study shows that study participants are primarily motivated by personal benefit when volunteering to participate in clinical studies. Whether these study participants had an integral understanding of the ICS is not clear.

## Background

A clinical study is an investigation that uses human subjects to contribute to knowledge that can be applied to benefit society. Clinical studies typically evaluate an intervention that is applied to study subjects. The intervention might be a drug, vaccine or therapeutic or surgical procedure [1]. Clinical studies are indispensable for the progress of medicine, especially for the discovery of new pharmaceuticals [2]. However, the use of human subjects in these studies as a unit of analysis introduces certain challenges [3].

In the past, study participants have suffered from the experiments that they were subjected to. One example is the experiments that were performed on prisoners during World War II [4], which generated a series of tragedies that are still remembered today. Those experiments violated the ethical rules that are currently applied to clinical studies [5,6].

Past violations have contributed to the development of mechanisms to protect human subjects [7]. The scientific community was made aware of these violations, and various regulations have been elaborated to protect human rights and the integrity and dignity of individuals participating in biomedical research [8].

However, the unethical studies of the past have left a strong impression and are still referenced in developing countries. In Brazil, the general perception is that the risks to study subjects are greater than the benefits, and the term “human guinea pig” is commonly used to describe clinical study participants [9].

The basic principles of bioethics (autonomy, beneficence, non-maleficence and justice) [10] may be violated in clinical studies on human subjects in such developing countries as Brazil and may be a source of bias within the study [11]. The characteristics of study populations from these countries, such as poverty, illiteracy, lack of education and lack of availability/access to health care, may create inherent bias. In addition, several authors warn that populations in developing countries require special protection, due to these factors [12]. These study subjects may not understand the study process or may participate in clinical studies because they do not have access to medical care. Other authors have noted that these study participants are more susceptible to coercion and may more easily become volunteers in clinical studies [13].

Fortunately, it is clear that there has been increased emphasis on bioethics in Brazil in relationship to clinical studies of human subjects. The Clinical Study Regulation was created under Resolution 196/96 of the National Health Council (Conselho Nacional de Saúde-CNS) of the Health Ministry. As a result, the ethical evaluation process that a clinical study must pass is sufficiently rigorous and is in accordance with national laws [14]. In addition to the bioethics evaluation performed by the Ethics in Research Committee (Comitê de Ética em Pesquisa - CEP) of the study institution, international studies must also receive consent from the National Commission of Ethics in Research of the National Health Council (Comissão Nacional de Ética em Pesquisa do Conselho Nacional de Saúde - CONEP) and the National Sanitary Monitoring Agency (Agência Nacional de Vigilância Sanitária - ANVISA) [5].

All subjects receive an Informed Consent Statement (ICS). This document explains in accessible language the research details to ensure that that the subject understands the procedures, risks, discomforts, benefits and rights involved and makes an autonomous decision [15,16].

Despite the rigor of these interventions, the idea persists that study subjects in Brazil participate in clinical studies for the wrong reasons [9].

The objective of the present study is to identify the specific motivators that inspire Brazilian volunteers to participate in clinical studies and to describe the demographic profile of these study subjects. In addition, we will evaluate whether the motivations are in accordance with established ethical and legal principles.

## Methods

### Rationale for the choice of methodology

A multi-methods study [17] with qualitative and quantitative phases was used. Qualitative methods were used to identify the motivations for study participation, focusing on interviewees perception and behaviors [18]. Quantitative analyzes were used to compare the motivators for research participation between groups and the variables that are associated with this motivation.

### Recruitment of the participants

A convenience sample was used. Three clinical research centers that conduct Phase I (bioequivalence) and Phase III (therapeutic) studies provided the records of subject participants. One center was in São Paulo, another was in Campinas and the third was in Goiania. In Brazil, there are few Phase I studies. Therefore, we considered bioequivalence studies, which use healthy volunteers to compare the bioavailability of a generic medicine to a reference medicine, to be Phase I studies. The participants were selected from 10 lists with a total of 500 volunteers each and were contacted by telephone for a personal interview in the Federal University of São Paulo (Universidade Federal de São Paulo, UNIFESP). These lists were composed of volunteers who participated in previous studies when they left permission to be contacted for future studies. They included the names of participants of studies conducted by the centers for the two years previously. The participation rate was 89%. In total, 90 participants were contacted (30 from each center), and 80 agreed to participate. The 10 refusals to participate included 4 men and 6 women, from the 3 centers.

### Procedure for translating the data

The interviews were conducted in Portuguese being recorded and later on transcribed and analyzed. The final result was translated into English by AJE (American Journal Experts) a site of experts from the scientific community with expertise in the two languages (English and Portuguese)*.* The quotes were back translated in order to ensure the fidelity of the interviewees’ statements.

### Sample size

The sample was composed of 80 volunteers who had participated in at least one Phase I or Phase III clinical study. The sample size was limited by the qualitative in-depth interview. Furthermore, this pilot study was not intended to be representative of the entire population of study participants. Rather, this study was designed to identify the main motivations that lead to clinical study participation and whether these motivators differ by research phase.

### Key informants (KI)

Key informants were people with specific knowledge regarding the study population and who were prepared to share knowledge about the study. The KI introduced the theme of the research, noting the peculiarities of the participants [19]. A total of ten KIs were interviewed. Three were principle investigators from the study centers, two were clinical study center coordinators, one was a representative of the Brazilian Society of Clinical Study Professionals (Sociedade Brasileira de Profissionais de Pesquisa Clinica-SBPPC), one was a representative of the Brazilian Association of Clinical Study Representative Organizations (Associação Brasileira de Organizações Representativas de Pesquisa Clínica-CRO), one was a regional director of clinical operations of the pharmaceutical industry and two were doctors responsible for conducting the studies in public and private reference hospitals. The interviews with the KIs were unstructured, conversational interviews [20,21]. They were recorded, transcribed, analyzed and provided information that could be used to develop interview questions for the clinical study subjects and to aid in understanding the discussions of the interviewed individuals [19].

These interviews allowed for comparative analysis of the motivations for participation in research studies according to subjects and KIs.

### Data collection instrument

The categories that emerged from the discussion with the KIs were used to describe the study subject volunteer profile and topics in the survey script. This survey script included sociodemographic data (age, gender and education), type of clinical study (Phase I or III), the involved pathology and location, the number of times that the volunteer participated in clinical studies, the motivation for participation, information concerning the ICS (whether it was introduced correctly and whether the volunteer could explain the study topic and remember the ICS), compensation received for participation and recruiting vehicles (how the volunteer found out about the study). Socioeconomic class was measured using the ABEP scale (Brazilian Association of Research Companies - Associação Brasileira de Empresas de Pesquisa), which takes into consideration the education level of the head of the household and ownership of assets [22]. This scale was used to sort participants into standardized subgroups labeled from A to E where A was the highest economic strata. Mean family income (MFI) at the E-level of the ABEP index is very low (below a 'living wage' level considered acceptable for families in the US), whereas a D-level family enjoys a MFI value roughly 1.5 times the E-level MFI. The C-level MFI is roughly 2–3 times the E-level value, A-level and B-level is approximately 27 times and 9 times the E-level MFI value respectively.

The anonymous interviews were semi-structured, in-depth conversations that were recorded with the consent of the respondent. The interviews were approximately 40 minutes in duration, and the participants were compensated R$20.00 (twenty reais) upon completion of the interview.

### Analysis of the results

#### Qualitative analysis of the interviews

After transcription, the interviews were submitted for content analysis, as proposed by Taylor and Bogdan [23] and Bardin [24] in accordance with the following steps:

### Initial reading

The initial reading allowed for the formation of general impressions.

### Preparation of the material

The material was prepared by separating and categorizing the responses according to topic. This material gave rise to independent archives for each script item: age, gender, education level, motivation for participation, study type, number of times the individual participated in clinical studies, method of recruitment, compensation for participation, understanding of the ICS and socioeconomic class. Each item included 80 responses that correspond to each sample constituent. With this information, the categories were constructed.

### Treatment of the results

The frequencies were calculated, allowing for interpretation. In the present study the triangulation technique was used [19]. Each interview was coded by more than one researcher to ensure the consistency of interpretations and increase the reliability of the categories. Excerpts from the interviews with KIs and study subjects appear in italics in the results.

### Statistical analysis

Hypothesis tests were conducted for each of the script variables to compare the characteristics and motivations of Phase I and Phase III study subjects. After codifying the answers from the qualitative phase, each theme was analyzed to generate up to 5 response categories. These variables were tested for the homogeneity of the distribution of responses across categories. The Student’s t-test was used to evaluate average age, study phase and research motivation. The Pearson’s chi-square test or the Fisher’s exact test was used for the categorical variables. A significance level of 5% was adopted, and Stata version 11 software was used to perform the analyses.

### Ethics

The protocol was reviewed and approved by the *Universidade Federal de São Paulo* (UNIFESP) Research Ethics Committee (Protocol #0870/10), with provisions for participants to participate anonymously, to decline to participate, to leave questions unanswered, and that they could interrupt their participation at any time, according to the Declaration of Helsinki. No one of the researchers occupy dual role (they were not clinicians or clinical researcher’s managers).

## Results

There was a predominance of women among the respondents (58%). Table 1 shows that the majority completed high school or college (approximately 90%), were less than 50 years of age (72%), were in either socioeconomic class B or C (84%) and had participated in only one clinical study (63%).

**Table 1:** Sociodemographic data for the 80 respondents according to research phase

**Characteristics**	**Phase I N** (**%)**	**Phase III N (%)**	**TOTAL N**
**Gender**			
Male	19 (47.5)	27 (67.5)	46
Female	21 (52.5)	13 (32.5)	34
**Education**			
Primary	4 (10.0)	4 (10.0)	8
High School	17 (42.5)	18 (45.0)	35
College	18 (45.0)	18 (45.0)	36
NR	1 (2.5)	0 (0)	1
**Socioeconomic class**^**1**^			
A	0 (0)	0 (0)	0
B	11 (27.5)	4 (10.0)	15
C	28 (70.0)	24 (60.0)	52
D	1 (2.5)	9 (22.5)	10
E	0 (0)	3 (7.5)	3
**Number of studies**			
1	24 (60.0)	26 (65.0)	50
2	1 (2.5)	11 (27.5)	12
3	9 (22.5)	2 (5.0)	11
4 or more	6 (15.0)	1 (2.5)	7
**Age (years)**			
≤ 20	1 (2.5)	0 (0)	1
21 - 30	19 (47.5)	1 (2.5)	20
31 - 40	11 (27.5)	4 (10.0)	15
41 - 50	8 (20.0)	14 (35.0)	22
51 - 60	0 (0)	9 (22.5)	9
61 or more	0 (0)	12 (30.0)	12
NR	1 (2.5)	0 (0)	1

*NR*= No Response.

^**1**^**A **was the highest economic strata. Mean family income (MFI) at the E-level of the ABEP index is very low (below a 'living wage' level considered acceptable for families in the US), whereas a **D-**level family enjoys a MFI value roughly 1.5 times the **E**-level MFI. The **C**-level MFI is roughly 2–3 times the **E**-level value, A-level and B-level is approximately 27 times and 9 times the E-level MFI value respectively.

### Analysis of the interview

#### Motivation to participate

**Figure 1:**
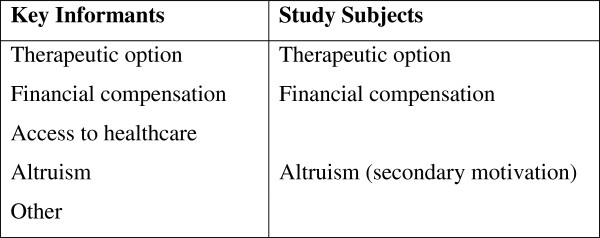
Principal motivators for participation in a clinical study according to KIs and study subjects.

Content analysis of the interviews identified motivating factors for participation in clinical studies. Figure 1 lists the main motivation for participation categories that emerged from the discussions with the KIs and study subjects. Both groups identified the therapeutic option and financial compensation as key motivators, suggesting that the KIs understood the motivations of the volunteers.

### Therapeutic option

#### Key informants

Opinion of the KIs versus the study subjects

According to the KIs, the therapeutic option to have access to a new treatment and the hope that this new drug can produce an improvement is a motivator to engage in clinical studies for potential experimental treatments. The discussions with the KIs in the following transcripts reveal the opinions of these professionals.

The group of patients, because they already have the illness, is always searching for something better, a new treatment. They submit themselves to the collateral effects of the medication to try a new medication, i.e., for cancer.

Others opt to participate in the study because they do not have another therapeutic option. These cases are more common in oncology and rare illnesses with few conventional treatment options.

### Study subjects

Motivation for participating in a clinical study according to study participants:

According to study subjects, the therapeutic option is a strong motivator to enroll in a clinical study. The potential benefit of a new medication was the most cited motivator. However, it is worth noting that this motivator is restricted to those volunteers with some type of pathology (in this case, Phase III study subjects). The following excerpt is from an interview with a study participant:

Motivation? It was for the benefit itself because it was a problem that I have. I thought: let’s do it!

### Financial compensation

#### Key informants

According to the KIs, financial compensation influences a volunteer’s decision to participate in a clinical study, especially among healthy volunteers. Although the Brazilian legislation only allows for compensation for time spent in the study, this remuneration is often attractive to a portion of the participants:

I believe that the healthy volunteers, in Phase I studies and in some other situations are motivated by a financial compensation, although in accordance with resolution 196 we cannot pay a Study Subject in Brazil, but in some way this compensation is the greatest motivation to participate in the study.

There is a camouflaged payment [compensation for the work-loss day], but many times it is much higher that the workday compensation.

### Study subjects

Healthy volunteers identified compensation as the principal motivation for participation in a study. The majority of volunteers did not want to reveal the amount that they received for participating in these studies.

It is not the first time that I have participated. They pay well, and I have confidence in the place and it is good because they do several examinations, and I a find out about how my health is.

### Access to healthcare

#### Key informants

According to KIs, many study subjects enter clinical studies because they believe that they will have improved access to health care and better quality of care. The subjects believe that they will be better able to check up on their health and will be able to avoid the time that they would spend in the public health sector. Moreover, the ability to call a study physician at any time is attractive to these patients.

In general the people, who participate of clinical studies, participate in research projects because they receive excellent attention and a healthcare option that is frequently better than the healthcare medical plans.

The patient feels truly taken care of, this is very difficult for me to assume, but it is what happens, the easiness in the attendance, the patients undergo examinations, they are taken care of with a special attention by the doctor…

The KIs mentioned *access to healthcare* as one of the main reasons for engaging in a clinical trial. However, the clinical trial participants did not evaluate it the same way. They acknowledge *access to healthcare* as a benefit but secondarily to other reasons that they consider more important for participating in a clinical trial, such as: financial advantage or therapeutic option.

### Altruism

#### Key informants

The KIs believed that a small portion of the study subjects altruistically engage in these studies to improve the lives of others.

I believe that there is an emotional component to this condition that emerges. For example, a father that volunteers for an AIDS vaccine because he lost a child to the disease.

### Study subjects

Some of the respondents mentioned the possibility of helping other people as a motivator for participating but always as secondary motivation.

It was to improve my knee and also to help in the study.

*First, because I have arthritis, and my healthcare plan was very bad….* and *second, because I am helping the researcher, and who knows, maybe humanity.*

### Other motivations

#### Key informants

The KIs also commented that the possibility for volunteers to make friends with people of a similar age and health status might lead them to participate.

(…) The elderly women created a certain community then, they met in the waiting room, they exchanged experiences, they talked and they started to meet outside of the clinic.

The possibility of receiving the test medication for the entire illness period, even after completing the study, was another motivation that was mentioned by the KIs.

### Study subjects

Few study subjects cited motivations other than a new therapeutic option and financial compensation. However, some added additional motivators, such as the recommendation of their personal physician to participate in the study.

I wanted to try another treatment. The ones that I had tried had no effect. That was when my doctor said that there might be a good opportunity for me to participate in a study for a new arthritis medication.

### Informed Consent Statement (ICS)

All the interviewed subjects declared that they had read and understood the ICS. They also reported that when they were unsure that there was a professional available to help them. However, when asked if they could remember the principal points of the ICS, a number of participants did not remember any of the content. Among those that remembered some parts of the ICS, none mentioned the potential risks of the study in which they participated.

That I could quit whenever I wanted to, and that I could speak with a doctor at any time.

I could stop when I wanted to, that I would take laboratory medicine with a name that I can’t remember…I had to do the exercises correctly.

I gone through the entire interview, I did all the examinations, but I do not remember what was written, I enrolled with the desire to resolve this [health] problem.

### Results of the statistical analysis

Table 2 shows that participants in different types of studies have different motivations to participate (p < 0.0001). For example, 94.7% of respondents that were motivated by financial compensation were Phase I study subjects. Conversely, 100% of the respondents who were searching for a therapeutic option were Phase III study subjects.

**Table 2:** M**otivators for clinical study participation according to study phase (N=80)**

**Motivators**	**Phase I (n = 40)**	**Phase III (n = 40)**	
	**n (%)**	**n (%)**	**P - value**^**2**^
			< 0.0001
Financial Compensation	36 (94.7)	2 (5.3)	
Therapeutic option	0 (0.0)	34 (100.0)	
Other^1^	4 (50.0)	4 (50.0)	

^1 ^Including altruism, knowledge, medical indication and access to healthcare; ^2 ^Fisher exact test.

Other motivations such as altruism and access to the healthcare were mentioned but were not the main motivators. Only 4% of participants alleged that these were the principal motivators.

The average age of participants (Table 3) was significantly different depending on the phase of the clinical study (p <0.0001). Those that participated in Phase I studies were younger (average age: 32 years; 95% CI: 28.7-34.8) than participants in Phase III studies (average age: 53 years: 95% CI: 49.1 - 56.0%).

**Table 3:** Average ages of the study subjects, according to research phase and motivator (N=79)

	**N**	**Age (average)**	**CI 95%**	**P-value**^**1**^
				<0.0001
Phase I	39	32	28.7 - 34.8	
Phase III	40	53	49.1 - 56.0	

Using Student’s t-test.

The average age varied by study purpose (p < 0.0001). The bioequivalence and medication studies had the youngest average ages (average age: 31.5; SD= 9.3). In this case, all were phase I study subjects (Table 4).

**Table 4:** Average age, according to type of study (N=79)

**Type of study**	**N**	**Age (average)**	**Standard deviation**	**p-value**^**2**^
				**<**0.0001
Arthritis/osteoarthritis	24	56.7	8.4	
Bioequivalence	39	31.5	9.3	
Insomnia	10	48.2	12.4	
Other^1^	6	43.3	9.9	

Included studies of menopause and temporomandibular pain;

ANOVA.

Most of the volunteers from both phases belonged to class C. However, the proportion of class D and E subjects varied according to study phase. Only 2.5% of participants from Phase I clinical studies belonged to the classes D and E. None of the respondents belonged to class A (Table 5).

**Table 5:** Socioeconomic class (based on the ABEP scale), stratified by study phase (N=80)

**Socioeconomic class**	**Phase I (n=40)**	**Phase III (n=40)**	
	**n (%)**	**n (%)**	**p-value**^**1**^
A	0 (0)	0 (0)	< 0.0005
B	11 [25]	4 [10]	
C	28 (70)	24 (60)	
D	1 (2.5)	9 (22.5)	
E	0 (0)	3 (7.5)	

^**1 **^Fisher’s exact test.

No statistically significant differences in the proportions of men to women when stratifying for study purpose or research phase. In addition, there were no differences in social class or education level when stratified by motivation for entering the study (data not shown).

## Discussion

Developing nations are participating in multi-center clinical studies at increasing rates. This participation is due to the reduced operational costs, ease of recruiting study subjects, ability to conduct research and regulatory capacity of these countries [26]. According to several authors, this shift is primarily due to economic considerations [20,27], suggesting that volunteers for clinical studies in developing countries are “guinea pigs” [9]. Therefore, there should be heightened concern for the ethical requirements to conduct a clinical study in these areas [21]. Further innovation in the pharmaceutical field requires these countries to be able to conduct clinical studies ethically. It is essential for international studies to be conducted in Brazil to advance the body of knowledge [2].

Despite advances in legislation and in professional development in this field, Brazil is far from being considered a major research center. The idea that Brazilian research participants are guinea pigs is not justified. Brazil conducts only 1.8% of total global clinical studies (n =1417). In contrast, the United States performs the majority of clinical studies; 54.3% of global clinical studies are conducted in the US (n= 41917) [25]^.^

These numbers demonstrate that Brazil is still not an important clinical research center, despite having characteristics that would be conducive for performing studies. Therefore, it is inappropriate to accuse Brazil of being the supplier of guinea pigs for clinical studies [9]. However, this finding should not decrease the focus on ethical concerns for clinical studies. These advances must be made rationally and should avoid unfounded critiques and prejudices of emerging countries.

Altruism should be the main reason for a subject’s decision to participate in a research study^28)^. Ideally, the volunteer is capable of making decisions based on the information provided about the proposed study, and understands the purpose, risks, benefits, alternatives and requirements of the study. After receiving this information, the volunteer is able to decide to participate, free from coercion or improper influences [28]. However, there is concern that the benefits may interfere with the study subject’s evaluation of the risks [21].

The results of the present study, although preliminary, show that health and financial benefits are the primary motivators for respondents to become study subjects. Individuals from higher socioeconomic classes were more likely to cite altruism as a secondary motivator after economic advantage (Phase I) or therapeutic option (Phase III). The same motivations were also identified by the KIs.

Although several authors insist that these motivators are more common in developing countries[21], studies demonstrate that they are found in study subjects throughout the world [29,30].

In a review by Stunkel and Grady [29], 12 of 13 studies showed that financial motivation was the main reason to participate in studies. One volunteer from these studies comments, “*Nobody is Robin Hood to make the good for the society*.” In the studies where the participants affirmed that compensation was the main motivator, no participants refused payment [31].

In a study of the motivations for healthy subjects to participate in research Vrhovac et al. [32] found that almost 80% reported compensation as the primary motivator. However, in that study, 20.6% of the volunteers denied that the money was the principal reason and identified humanitarian motivations and the desire to contribute to society. The key informants for the present study also mentioned altruism; however, it was only mentioned in exceptional situations.

Alternatively, there are study subjects that believe that compensation should be commensurate with the risks of the study and that compensation should be increased for invasive procedures, such as drawing blood [33].

Almeida et al. [34] found that volunteers with low education and low socioeconomic status were more likely to cite financial motivations to participate in a clinical study.

In this study, we did not find this association; there was no difference in education level between Phase I and Phase III participants. However, participants in a higher socioeconomic class were more likely to participate in Phase I studies (Table 5) (thus, according to Table 1, they were more likely to be healthy volunteers participating for financial reasons). These findings are in agreement with those of Kass et al. [30], who found that Caucasians with a higher education level were more likely to be motivated by financial benefits. These findings are in contrast to the beliefs of some authors who say that compensation attracts low-income volunteers, resulting in a disproportionate number of poor people participating in clinical studies [35].

Gelderen [36] found that the youngest volunteers were the most likely to mention money as the primary motivator to participate, which is a finding that was reproduced in our study (average age of 32 years in Phase I participants). This result is not surprising, as younger people might not be as financially established and may be looking for ways to earn money. Moreover, Phase I participants are required to be healthy, which is a criterion that is more common among younger adults.

Among Phase III participants, the principal motivation for participation in clinical studies was to search for a new treatment, a motivator that was also mentioned by the KIs. Grecco and Diniz [37] have noted that clinical study volunteers in developing countries see participation in clinical studies as an opportunity to receive better healthcare, easier access to more expensive laboratory examinations and novel drugs. According to Grecco and Diniz, these motivations constitute a conflict of interests. It is worth noting that Brazilians have universal access to the Unified Healthcare System (Sistema Único de Saúde – SUS), which is funded by the federal government [38]. However, access to this system does not limit the interest in clinical studies, perhaps due to the quality of the services or the degree of individualized attention.

Cabral et al. [1] have noted that in the research environment, the physician-patient relationship is inverted. Classically, the physician satisfies the needs and interests of the patient. However, in a clinical study, the patient satisfies the interests of the study and thus the researcher. Lackey [27] adds that the researcher, for the advancement of the study, must treat these subjects as scientific objects submitted to the laws of cause and effect. The study subjects may not understand this distinction and may continue to participate in the study expecting a cure when, in reality, the medicine may not have any effect.

Canvin and Jacob [39] observed a group of volunteers for a study of epilepsy and noted that these volunteers were happy to help others only when they could also help themselves. The authors called this behavior “weak altruism,” and McCann et al. [40] have called this behavior “conditional altruism.” According to these authors, the desire to help others or to contribute to the body of knowledge does not lead a volunteer to participate in a study unless that volunteer perceives that his participation in the study may benefit him personally.

The Informed Consent Statement (ICS), which is required under Resolution 196/96 (CNS/196), is provided for the volunteer to inform him about the study, including the associated risks and the responsibilities of those involved. The ICS is indispensable to the ethical conduct of the research. According to a number of authors, volunteers do not always pay enough attention to the ICS. This oversight may occur because the volunteer trusts the doctor (or at least the role of the doctor) and does not take into consideration the role of the doctor as a researcher, that is, the doctor is not necessarily focused on meeting the patient’s global health needs [27]. Additionally, the patient may overestimate the benefit for himself and lose the ability to weigh the disadvantages of the study [41]. Finally, the volunteer may not understand the ICS because of a low educational level [42]. In the present study, most volunteers had a good education, and all declared that they understood the ICS. However, when they were questioned about the study details, they only remembered the possibility of *speaking with a doctor* and the possibility of *leaving the study.* None of the subjects mentioned the disadvantages of the study, such as the risks or potential adverse reactions to the medicine. These findings may indicate that the volunteers did not adequately understand the ICS. It is worth noting that a portion of the respondents did not remember that some items of ICS may be limited to the time that they participated in the study. The more “comfortable” situations associated with the study were remembered, while the situations associated with risks were not mentioned. However, the date of last participation in a clinical trial was not questioned, which may constitute a limitation in analysis of the data.

## Conclusion

In the present study, study volunteers were motivated by some type of personal benefit from participating in a study (especially financial or therapeutic benefits), which is a finding has been observed in clinical study participants in other countries. According to several authors, this finding is accentuated in emerging countries, such as Brazil, due to the limitations of the health care systems, even despite the universal access provided by the SUS. Altruism was not a common motivator, and when it was mentioned, it was never the primary reason. Some have called type of motivation this “conditional altruism”. The ICS was understood by all participants; however, the degree of understanding may have been limited. None of the subjects remembered the text referring to the potential harms of the procedure or medicine. The authors hypothesize that this behavior results because the individuals participate out of self-interest, rather than altruism.

### Study limitations

This study was a preliminary study with a qualitative-quantitative approach. The study sampled 80 participants and was not representative of the total population of clinical study participants. Another limitation is the fact that those who refused to participate in the research were not surveyed.

### Study strengths

This study demonstrates that personal benefit is the primary motivation for volunteers to participate in clinical research. However, we argue that this motivation seems to be universal and is not limited to developing countries. Moreover, the theory that financial compensation disproportionately attracts poor volunteers has not been supported. Furthermore, the fact that study participants are primarily motivated by personal benefit does not suggest that they do not evaluate the risks of participation. For further consideration, if we consider altruism to be the only ethically acceptable motivation for study participation, must we evaluate whether the principle of autonomy has been disrespected?

## Competing interests

The authors declare that they have no competing interests.

## Authors' contributions

SAN conceived the study and participated in its design and coordination and wrote the final version of the manuscript. GBI collected data and ZMS performed the statistical analysis and interpreted the data obtained. All authors read and approved the final manuscript.

## Pre-publication history

The pre-publication history for this paper can be accessed here:

http://www.biomedcentral.com/1471-2458/13/19/prepub

## References

[B1] Cabral MML Schindler HC Abath FG Regulamentações, conflitos e ética da pesquisa médica em países em desenvolvimento Rev Saude Publica 2006 40 3 521 527 10.1590/S0034-89102006000300022 16810378

[B2] Quental C Salles Filho S Ensaios Clínicos: capacitação nacional para avaliação de medicamentos e vacinas Rev Bras Epidemiol 2006 9 4 408 424 10.1590/S1415-790X2006000400002

[B3] Grecco D Diniz NM Conflicts of interest in research involving human beings J Int Bioethique 2008 19 1–2 143–54 61 18664007 10.3917/jib.191.0143

[B4] Garrafa V Lorenzo C Moral imperialism and multi-centric clinical trials in peripheral countries Cad Saude Publica 2008 24 10 2219 2226 10.1590/S0102-311X2008001000003 18949224

[B5] CNS -Conselho Nacional de Saúde Resolução 196 de outubro de 1996 1996 Disponível em: http://conselho.saude.gov.br/resolucoes/reso_96.htm (last accessed 04/07/12)

[B6] WHO Ethics in international health research: a perspective from the developing world Bull World Health Organ 2002 80 114 120 11953789 PMC2567726

[B7] Editorial Ethical behavior in clinical research- a lesson from the past Lancet 2011 378 9795 962 10.1016/S0140-6736(11)61433-5 21907844

[B8] Garrafa V Prado MM Mudanças na Declaração de Helsinki: fundamentalismo econômico, imperialismo ético e controle social Cad Saude Publica 2001 17 6 1489 1496 10.1590/S0102-311X2001000600033 11784910

[B9] Lacativa PGS Szrajbman M Silva DASM Melazzi ACC Gregório LH Russo LAT Perfil de sujeitos de pesquisa clínica em um centro ambulatorial independente Cad Saude Publica 2008 13 3 1023 1032 10.1590/s1413-81232008000300025 18813596

[B10] Beauchamp TL Childress JF Principles of Biomedical Ethics 1994 4a New York: Oxford University Press

[B11] Zegers-Hochschild F Barriers to conducting clinical research in reproductive medicine: Latin America Fertil Steril 2011 96 4 802 804 10.1016/j.fertnstert.2011.08.043 21961912

[B12] Shamoo AE Katzel LI Urgent Ethical Challenges in Human Subjects Protection J Clin Res Best Pract 2007 3 3 1 5

[B13] Farrell K Human experimentation in developing countries: improving international practices by identifying vulnerable populations and allocating fair benefits J Health Care Law Policy 2006 9 1 136 161 17165228

[B14] Marodin G Goldin JR Confusões e ambigüidades na classificação de eventos adversos em pesquisa clínica Rev Esc Enfer USP 2009 43 3 690 696 10.1590/S0080-62342009000300027 19842604

[B15] Goldim JR Pithan CF Oliveira JG Raymundo MM O processo de consentimento livre e esclarecido em pesquisa: uma nova abordagem Rev Assoc Med Bras 2003 49 4 372 374 10.1590/S0104-42302003000400026 14963587

[B16] Mueller MR Instone S Beyond the informed consent procedure: continuing consent in human research Cad Saude Publica 2008 13 2 381 389 10.1590/s1413-81232008000200013 18813554

[B17] Creswell JW Research design: Qualitative, quantitative and mixed methods approaches 2009 3 USA: Sage Publications

[B18] Minayo MCS O desafio do conhecimento: pesquisa qualitativa em saúde 1994 São Paulo: RJ:HUCITEC-ABRASCO

[B19] Patton MQ Qualitative research and evaluation methods 2002 USA: Sage Publications

[B20] Castilho EA Kalil J Ética e pesquisa médica: princípios, diretrizes e regulamentações Rev Soc Bras Med Trop 2005 38 4 344 347 10.1590/S0037-86822005000400013 16082484

[B21] Shah JY Phadtare A Rajgor D Vaghasia M Pradhan S Zelko H Pietrobon R What Leads Indians to Participate in Clinical Trials? A Meta-Analysis of Qualitative Studies PLoS One 2010 5 5 e10730 10.1371/journal.pone.0010730 20505754 PMC2873955

[B22] ABEP Associação Brasileira de Empresas de Pesquisa - Critério de Classificação Econômica Brasil] 2010 Brazil: Brazilian Association of Research Firms - Criteria for Economic Classification Brazil [Internet]. Available from: www.abeporg/codigosguias/Criterio_Brasil_2008pdf 2008

[B23] Taylor S Bodgan R Introduction to Qualitative Research Methods 1998 New York: John Wiley & Sons Inc.

[B24] Bardin L Análise de Conteúdo 2004 3ª Edições 70, Lisboa

[B25] NIH – National Institute of Health, USA Search for Clinical Trials Disponível em: http://www.clinicaltrials.gov (last accessed 04/07/2012)

[B26] Thiers FA Sinskey AJ Berndt ER Trends in the globalization of clinical trials Nat Rev Drug Discov (on-line) 2007 November, 2

[B27] Lackey DP Critical Research in developing countries recent moral arguments Cad Saude Publica 2002 18 5 1455 1461 10.1590/S0102-311X2002000500038 12244378

[B28] Grady C Money for Research Participation: Does it jeopardize informed consent? Am J Bioeth 2001 1 2 40 44 10.1162/152651601300169031 11951886

[B29] Stunkel L Grady C More than the money: A review of the literature examining healthy volunteer motivations Contemp Clin Trials 2011 32 342 352 10.1016/j.cct.2010.12.003 21146635 PMC4943215

[B30] Kass NE Myers R Fuchs EJ Carson KA Flexner C Balancing justice and autonomy in clinical research with healthy volunterers Clin Pharmacol Ther 2007 82 2 219 227 10.1038/sj.clpt.6100192 17410122

[B31] Mtunthama N Malamba R French N Molyneux ME Zijlstra EE Malawians permit research bronchoscopy due to perceived need for healthcare J Med Ethics 2008 34 4 303 307 10.1136/jme.2007.020461 18375686

[B32] Vrhovac R Francetic I Rotim K Drug trials on healthy volunteers in Yugoslavia Int J Clin Pharmacol Ther Toxicol 1990 28 9 375 2228323

[B33] Fergson PR Clinical trials and healthy volunteers Med Law Rev 2008 16 1 23 51 10.1093/medlaw/fwm020 18174206

[B34] Almeida L Azevedo B Nunes T Vaz-da-Silva M Soares-da-Silva P Why healthy subjects volunteer for phase I studies and how they perceive their participation? Eur J Clin Pharmacol 2007 63 11 1085 1094 10.1007/s00228-007-0368-3 17891536

[B35] Stones M McMillan J Payment for participation in research: a pursuit for the poor? J Med Ethics 2010 36 1 34 36 10.1136/jme.2009.030965 20026691

[B36] Gelderen CEM Savelkoult TJF Dokkum W Meulenbelt TJ Motives and perception of healthy volunteers who participate in experiments Eur J Clin Pharmacol 1993 45 1 15 21 10.1007/BF00315344 8405024

[B37] Greco D Diniz NM Conflicts of interest in research involving human beings J Int Bioethique 2008 19 1–2 143–54 202–3 18664007 10.3917/jib.191.0143

[B38] Victora CG Barreto ML Leal MC Monteiro CA Schmidt MI Paim J Bastos FI Almeida C Bahia L Travassos C Reichenheim M Barros FC The Lancet Brazil Series Working Group 2011 Disponível em: http://www.thelancet.com (last accessed 04/07/12)

[B39] Cavin K Jacoby A Duty, desire or indifference? A qualitative study of patient decisions about recruitment to an epilepsy treatment trial Trials 2006 7 32 10.1186/1745-6215-7-32 17163988 PMC1770934

[B40] McCann S Campbell MK Entwistle Reasons for participating in randomised controlled trials: conditional altruism and considerations for self Trials 2010 11 13 20307273 10.1186/1745-6215-11-31 PMC2848220

[B41] Lynöe N Sandlund M Jacobsson L Informed consent in two Swedish prisons: a study of quality of information and reasons for participating in a clinical trial Med Law 2001 20 4 519 523 11817382

[B42] Meneguin S Zoboli ELCP Domingues RZL Nobre MR Cesar LAM Entendimento do Termo de Consentimento pelos pacientes partícipes em pesquisas com fármacos de cardiologia Arq Bras Cardiol 2010 94 1 4 9 10.1590/S0066-782X2010000100003 20414520

